# Two to Tango: lipoprotein(a) and hormonal status as determinants of venous thromboembolism risk

**DOI:** 10.1093/eurheartj/ehag263

**Published:** 2026-03-28

**Authors:** Pam R Taub, Harpreet S Bhatia

**Affiliations:** Division of Cardiovascular Medicine, University of California-San Diego, 9300 Campus Point Drive #7414, La Jolla, CA 92037, USA; Division of Cardiovascular Medicine, University of California-San Diego, 9300 Campus Point Drive #7414, La Jolla, CA 92037, USA

## Abstract

**Graphical Abstract:**
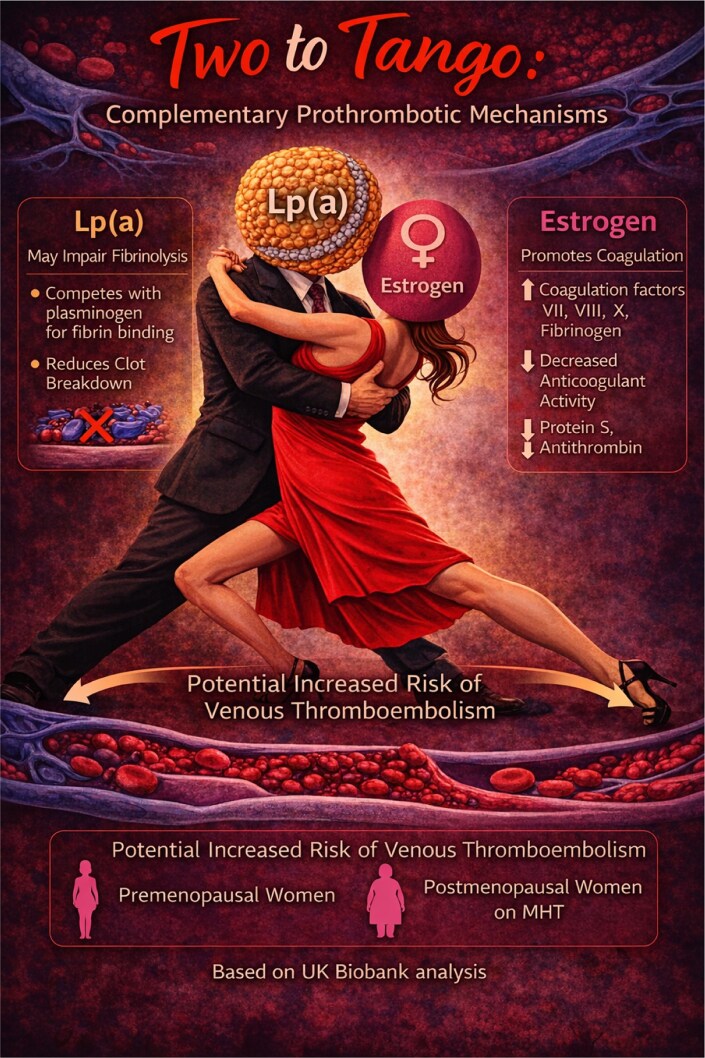
Complementary prothrombotic mechanisms of lipoprotein(a) [Lp(a)] and oestrogen. Oestrogen increases venous thromboembolism (VTE) risk through interactions with the coagulation system. Additionally, Lp(a) has proposed anti-fibrinolytic mechanisms, primarily demonstrated in *in vitro* studies. The present study, based on data from the UK Biobank, suggests that Lp(a) is associated with increased VTE risk in the setting of increased circulating oestrogen, either in premenopausal women or postmenopausal women on hormone replacement therapy. The figure illustrates a potential “two-hit” interaction between oestrogen and Lp(a), which may be associated with increased risk of VTE. Figure designed by the authors using an AI tool.

Complementary prothrombotic mechanisms of lipoprotein(a) [Lp(a)] and oestrogen. Oestrogen increases venous thromboembolism (VTE) risk through interactions with the coagulation system. Additionally, Lp(a) has proposed anti-fibrinolytic mechanisms, primarily demonstrated in *in vitro* studies. The present study, based on data from the UK Biobank, suggests that Lp(a) is associated with increased VTE risk in the setting of increased circulating oestrogen, either in premenopausal women or postmenopausal women on hormone replacement therapy. The figure illustrates a potential “two-hit” interaction between oestrogen and Lp(a), which may be associated with increased risk of VTE. Figure designed by the authors using an AI tool.

**This editorial refers to ‘Lipoprotein(a) and incident venous thromboembolism in pre- and postmenopausal women, and in men’, by D. Ezzat *et al*., https://doi.org/10.1093/eurheartj/ehag252.**

Over the past three decades, a robust body of evidence has established lipoprotein(a) [Lp(a)] as a causal risk factor for both atherosclerotic cardiovascular disease (ASCVD) and calcific aortic valve disease.^1^ Both of these conditions occur within the milieu of the arterial vasculature, where complex and dynamic interactions take place between atherosclerotic/calcific plaque, immune cells, and the thrombosis and coagulation pathways. Primarily *in vitro* studies have proposed antifibrinolytic mechanisms associated with Lp(a). Additionally, Lp(a) has been associated with potential proplatelet effects.^2^ However, there has not been a consistent association between Lp(a) and venous thromboembolism (VTE) risk in clinical settings. Studies evaluating both *LPA* genetic and plasma Lp(a) levels, including a prior UK Biobank study, have generally failed to demonstrate an association with VTE,^3–5^ though one study did observe an association between extremely high Lp(a) (>95th percentile) and VTE.^4^ Additionally, a potent reduction in Lp(a) with the targeted therapy pelacarsen was not associated with a change in clot generation or lysis time.^6^ Thus, *in vitro* findings have not consistently translated into clinical events. However, Lp(a) has not been examined in detail in conjunction with other prothrombotic risk factors, which raises the question: does Lp(a) contribute to VTE in specific subgroups with a predisposition for VTE, such as smokers and those on oestrogen-based hormonal therapy?

In this issue of the *European Heart Journal*, Ezzat and colleagues address this question by examining the association between Lp(a) and incident VTE in 373 360 participants from the UK Biobank followed for a median of 13.6 years.^7^ This population was categorized into three distinct hormonal environments: pre-menopausal women (55 302) with higher endogenous oestrogen levels, post-menopausal women with reduced oestrogen levels (129 045), and men (189 013). They further stratified these groups based on exogenous oestrogen exposure: pre-menopausal women on oral contraception (OCP) and post-menopausal women on menopausal hormone replacement therapy (MHT). Elevated Lp(a) >125 nmol/L was associated with a higher risk of incident VTE in pre-menopausal women (hazard ratio [HR] 1.32), but not in post-menopausal women or in men. In a subgroup analysis, however, post-menopausal women on MHT also had increased VTE risk, with a significant interaction between Lp(a) and MHT use.

These findings highlight the important interaction between Lp(a) and an individual’s hormonal environment. Oestrogen has prothrombotic effects, and both MHT and OCP, which increase oestrogen levels, are associated with increased VTE risk. This study suggests that Lp(a) may further amplify this hormonally mediated prothrombotic milieu. Interestingly, pre-menopausal women on OCP did not have increased risk of VTE, and this may be due to the limited number of events among 4800 OCP users and limited statistical power to detect interaction effects. In addition, information regarding specifics of OCP formulations was unavailable; there are many formulations that are progestin-only, and clinicians may avoid prescribing oestrogen-containing OCPs for women perceived to be at a higher thrombotic risk.

From a pathophysiological perspective, these observations from the UK Biobank are biologically plausible given the potentially complementary effects of oestrogen and Lp(a) on thrombosis pathways, and highlight the importance of Lp(a) within the context of an individual’s hormonal environment. Lp(a) is composed of apolipoprotein(a), a structural homologue of plasminogen, which competes with plasminogen for binding to fibrinogen and thereby may impair fibrinolysis. In parallel, oestrogen promotes a procoagulant state through increased synthesis of multiple clotting factors and reduced anticoagulant activity. Together, these processes may converge to create a ‘two-hit’ mechanism in which oestrogen promotes thrombus formation, while Lp(a) may impair thrombus degradation (*Graphical Abstract*). This has potential clinical implications for the use of MHT in women with elevated Lp(a), just as we would factor in family history of deep vein thrombosis (DVT)/pulmonary embolism (PE) and smoking history.

This study has notable strengths, including a large sample size with robust statistical methods as well as extensive sensitivity analysis, including modelling Lp(a) as a continuous variable, evaluating alternative thresholds for elevated Lp(a) and a genetic risk score for Lp(a), evaluating oestradiol levels, and accounting for confounders such as atrial fibrillation, which may prompt initiation of anticoagulation. However, these results should also be interpreted in the context of the inherent limitations of observational analyses, including limited data regarding OCP and MHT formulations and the use of diagnostic codes for outcome ascertainment. The number of VTE events in the overall cohort, and particularly within the subgroup analyses, was relatively low. Additionally, although there was an increase in relative risk, the absolute VTE risk remained low, even in pre-menopausal women. The replication of these findings in other cohorts, particularly those with greater ethnic diversity and more granularity, is warranted. Additionally, randomized clinical trials of widely available, inexpensive therapies such as aspirin would help determine whether targeted antithrombotic strategies mitigate the increased thrombotic risk associated with elevated Lp(a).

As we await the results of large cardiovascular outcome trials evaluating the impact of Lp(a)-lowering therapies on cardiovascular events, the study by Ezzat and colleagues highlights an important principle for the Lp(a) field in general: risk factors rarely operate in isolation but instead act within complex biological contexts.

## References

[1] Kronenberg F, Mora S, Stroes ESG, Ference BA, Arsenault BJ, Berglund L, et al Lipoprotein(a) in atherosclerotic cardiovascular disease and aortic stenosis: a European Atherosclerosis Society consensus statement. Eur Heart J 2022;43:3925–46. 10.1093/eurheartj/ehac361 36036785 PMC9639807

[2] Bhatia HS, Becker RC, Leibundgut G, Patel M, Lacaze P, Tonkin A, et al Lipoprotein(a), platelet function and cardiovascular disease. Nat Rev Cardiol 2024;21:299–311. 10.1038/s41569-023-00947-2 37938756 PMC11216952

[3] Helgadottir A, Gretarsdottir S, Thorleifsson G, Holm H, Patel RS, Gudnason T, et al Apolipoprotein(a) genetic sequence variants associated with systemic atherosclerosis and coronary atherosclerotic burden but not with venous thromboembolism. J Am Coll Cardiol 2012;60:722–9. 10.1016/j.jacc.2012.01.078 22898070

[4] Kamstrup PR, Tybjærg-Hansen A, Nordestgaard BG. Genetic evidence that lipoprotein(a) associates with atherosclerotic stenosis rather than venous thrombosis. Arterioscler Thromb Vasc Biol 2012;32:1732–41. 10.1161/ATVBAHA.112.248765 22516069

[5] Dentali F, Gessi V, Marcucci R, Gianni M, Grandi AM, Franchini M. Lipoprotein(a) as a risk factor for venous thromboembolism: a systematic review and meta-analysis of the literature. Semin Thromb Hemost 2017;43:614–20. 10.1055/s-0036-1598002 28346964

[6] Boffa MB, Marar TT, Yeang C, Viney NJ, Xia S, Witztum JL, et al Potent reduction of plasma lipoprotein (a) with an antisense oligonucleotide in human subjects does not affect ex vivo fibrinolysis. J Lipid Res 2019;60:2082–9. 10.1194/jlr.P094763 31551368 PMC6889713

[7] Ezzat D, Lopez DM, Claggett BL, Li L, Mohammadnia N, Schuermans A, et al Lipoprotein(a) and incident venous thromboembolism in pre- and post-menopausal women, and in men. Eur Heart J 2026;47:3576–87. 10.1093/eurheartj/ehag252 PMC13266305 41903523

